# 1998. The State of Training in Infection Prevention and Control and Healthcare Epidemiology: A National Survey of Infectious Disease Fellowship Program Directors

**DOI:** 10.1093/ofid/ofad500.125

**Published:** 2023-11-27

**Authors:** Marisa Winkler, Molly L Paras, Sharon B Wright, Erica S Shenoy

**Affiliations:** Emory University, Atlanta, GA; Massachusetts General Hospital, Harvard Medical School , Boston, MA; Beth Israel Lahey Health, Cambridge, Massachusetts; Massachusetts General Hospital, Boston, Massachusetts

## Abstract

**Background:**

Infectious disease (ID) clinicians are often identified to lead infection prevention and control (IPC)/healthcare epidemiology (HE) programs, however, there is no standardized training in fellowship for this role. The most recent survey of ID fellows in 2001 found that only 51% of graduates reported adequate training in IPC/HE. Despite this, half of respondents had IPC/HE responsibilities and received compensation from those activities. We conducted a survey of Program Directors (PD) of United States ID fellowship programs to assess current IPC/HE training.

**Methods:**

An electronic survey was distributed in March and April 2023 to all ID fellowship PD identified by the Infectious Disease Society of America using Research Electronic Data Capture (REDCap). Survey questions covered training program characteristics, current required and optional IPC/HE training components, barriers to IPC/HE training, interest among fellows in IPC/HE training, and support for formalization of IPC/HE training.

**Results:**

Of 166 programs, 54 (32.5%) completed the survey, representing programs in 25 states (Figure 1) and size range from 1-10 fellowship positions (mean 4). The majority (49/54, 90.7%) offered didactics in IPC/HE (mean 4.4 hours, range 1-10), of which 45/49 (94%) and 4/49 (6%) were required and elective, respectively. 52/54 (96.3%) of programs reported a formalized curriculum in IPC/HE training for all fellows and 18/54 (33.3%) of the responding programs offered a specialized track in IPC/HE. Reported training components varied (Table 1). A minority of PDs 11/55 (20%) reported considering expanding their IPC/HE training, however, 23/54 (42.6%) reported barriers (Table 2). There was widespread PD support for a formal IPC/HE certification program through a professional society (52/54, 96.3%).

**Figure 1:**
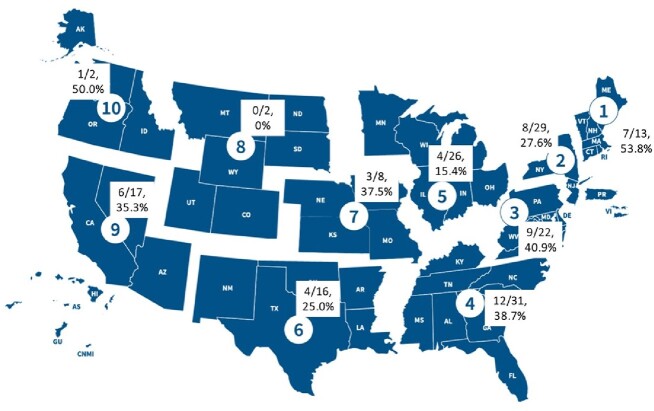
Geographic distribution of survey respondents divided by FEMA regions (1-10) of the United States and Territories (N= 54).

**Figure:**
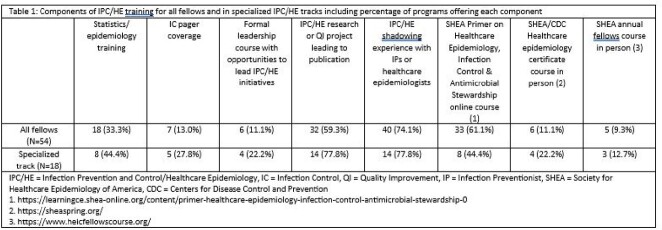

**Figure:**
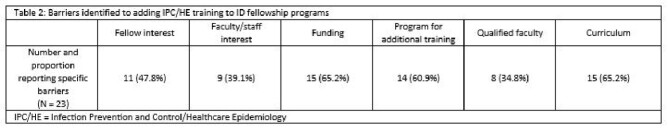

**Conclusion:**

ID fellowship programs with dedicated IPC/HE training remain limited and the majority of training in IPC/HE is restricted to a small number of hours of didactics. Among respondents, many barriers to increasing IPC/HE training were identified. Respondents supported creation of a formal IPC/HE training program through a professional society.

**Disclosures:**

**Molly L. Paras, MD**, Angiodynamics: Honoraria|Angiodynamics: Honoraria

